# Different vascular healing process between bioabsorbable polymer-coated everolimus-eluting stents versus bioresorbable vascular scaffolds via optical coherence tomography and coronary angioscopy (the ENHANCE study: ENdothelial Healing Assessment with Novel Coronary tEchnology)

**DOI:** 10.1007/s00380-019-01516-9

**Published:** 2019-10-05

**Authors:** Wan Azman Wan Ahmad, Takaharu Nakayoshi, Ahmad Syadi Mahmood Zuhdi, Muhammad Dzafir Ismail, Imran Zainal Abidin, Yasushi Ino, Takashi Kubo, Takashi Akasaka, Yoshihiro Fukumoto, Takafumi Ueno

**Affiliations:** 1 Division of Cardiology, Department of Medicine, University Malaya, Kuala Lumpur, Malaysia; 2 Department of Internal Medicine, Division of Cardiovascular Medicine, Kurume University School of Medicine, Kurume, Japan; 3 Division of Cardiovascular Medicine, Wakayama Medical University, Wakayama, Japan; 4 The Center of Cardiovascular Disease, Kurume University Hospital, 67 Asahi-machi, Kurume, 830-0011 Japan

**Keywords:** Vascular healing, Optical coherence tomography, Coronary angioscopy, Bioabsorbable polymer-coated everolimus-eluting stents, Bioresorbable vascular scaffolds

## Abstract

Recent clinical trials have raised concerns about the safety and efficacy of ABSORB™ bioresorbable vascular scaffolds (BVS). The difference in the vascular healing process between SYNERGY™ bioabsorbable polymer-coated everolimus-eluting stents (BP-EES) and BVS remains unclear. The aim of the ENHANCE study was to compare vascular healing on BP-EES versus BVS by optical coherence tomography (OCT) and coronary angioscopy (CAS) at 4- and 12-month follow-ups. This is a prospective, non-randomized, single center clinical trial. Thirteen eligible patients with multivessel disease were enrolled. BP-EES and BVS were simultaneously implanted in the same patients, but in different coronary vessels. Imaging follow-up with both OCT and CAS was completed in 11 patients at 12 months. Neointimal coverage rates were similar between the two groups based on OCT measurements. The neointimal thickness of BP-EES was significantly thicker at the 12th month than at the 4th month, whereas the neointimal thickness of BVS did not change between the measurements taken at the 4th and 12th month. Existence of intra-stent thrombus was significantly higher in the BVS group, compared to the BP-EES group. On the other hand, CAS revealed that red-thrombi and yellow-plaque were more frequently observed in BVS at 4 months and up to 12-month follow-ups than in BP-EES. These findings suggested that the evidence of instability remained up to 12 months in the vascular healing with BVS, compared to that with BP-EES. Vascular healing of the stented wall was recognized at the very early phase after BP-EES implantation. However, vascular healing with BVS was still incomplete after 12 months.

## Introduction

The drug eluting stent (DES) made it possible to drastically reduce restenosis, but some concerns regarding very late stent thrombosis have not yet been overcome. The delayed healing with incomplete neointimal coverage, persistent of inflammation, hypersensitivity and neoatherosclerosis at the stent implanted site may be involved in stent thrombosis [1–3].

The bioabsorbable polymer-coated everolimus-eluting Stent (BP-EES: SYNERGY™, Boston Scientific, Marlborough, MA, USA) was designed to enhance stent healing by incorporating thin PtCr struts with abluminal bioabsorbable polymer, which is reabsorbed within 4 months. These technical characteristics may promote a more complete and early vascular healing process after stenting, as has been suggested in animal models [4]. This stent has shown in trials a low late lumen loss and a clinical efficacy and safety profile not inferior to that of durable polymer-coated EES [5, 6]. Based on angioscopy findings, we reported on the process of vascular healing after implantation of BP-DES [7].

On the other hand, the bioresorbable vascular scaffolds (BVS: ABSORB™, Abbott Vascular, Santa Clara, CA, USA) have been developed in an attempt to improve long-term outcomes, followed by a novel approach for complete strut bioresorption over several years with late luminal enlargement [8]. However, the recent clinical trials of BVS presented unfavorable concerns about safety and efficacy regarding device and patient-oriented cardiac events [9]. The difference in vascular healing between BP-EES and BVS remains unclear. In particular, vascular healing after BVS deployment has, thus far, not been precisely evaluated with coronary angioscopy in vivo. In terms of polymer elution to the vessel wall, we hypothesized that BP-EES and BVS would have similar effects on the coronary vessel wall at an early stage, but may be different in the chronic stage.

The purpose of this ENHANCE study was to assess the vascular healing with BP-EES versus BVS in human coronary arteries by optical coherence tomography (OCT) and coronary angioscopy (CAS) at 4- and 12-month follow-ups.

## Methods

### Study population and lesion treatment

Assessment of vascular healing with BP-EES or BVS was a prospective, non-randomized, single-center clinical trial. From September 2015 to March 2016, a total of 13 patients with multivessel disease, who met all the following inclusion criteria and consented to the study protocol, were enrolled. BP-EES in one artery and BVS in another artery were simultaneously implanted in every patient at University Malaya Medical Centre, Kuala Lumpur, Malaysia.

#### Clinical criteria

1. Patients or their legally authorized representatives had to provide written informed consent prior to any study-related procedure, per site requirements.
2. Patients had to have evidence of myocardial ischemia suitable for elective PCI.
3. Patients had to present clinical characteristics ensuring feasible and angiographic follow-up safety (> 20 years, LVEF > 30%, not receiving hemodialysis).
4. Patients were eligible to undergo OCT and CAS examinations at 4 months and 12 months after stent or scaffold implantation.
5. Patients were able to take dual anti-platelet therapy up to 1 year following the index procedure and anticoagulants prior to/during the index procedure.

#### Angiographic inclusion criteria

Significant narrowing of at least 70% stenosis of de novo lesion (QCA or visual estimation) in two different native coronary arteries.

#### Angiographic exclusion criteria

1. Target vessels treated by PCI within 12 months.
2. Target lesions located in left main ostium, with TIMI flow 0 (total occlusion), highly tortuous equal to or greater than 60°, and within a saphenous vein graft or an arterial graft.

PCI was performed by the PSP method (pre-dilation, proper scaffold sizing, and post-dilation) [10] under OCT guidance. Stent or scaffold size and number were determined at the operator’s discretion. Adjuvant dilatation or additional stent or scaffold implantation were allowed and determined at the operator’s discretion. All patients were admitted for 1 day prior to the procedure and stayed overnight for monitoring before discharge.

The study was approved by the local research ethical committees. A written informed consent was obtained prior to all procedures from all patients in this study.

### Follow up examination

A 4- and 12-month angiographic follow-up was conducted using both OCT and CAS. The patient information, lesion characteristic information and QCA/OCT/CAS data were collected anonymously and analyzed in a core laboratory. QCA data was analyzed in an independent ENDO CORE laboratory at Fukuoka International College of Health and Welfare, Japan. OCT images were analyzed in an OCT core laboratory in Wakayama University, Japan and CAS images were analyzed in a CAS core laboratory in Kurume University, Japan. Both image data were analyzed by two independent analysts blinded to the clinical presentation and lesions’ characteristics, and the analysis was performed offline data, using proprietary software provided by Light Lab Imaging Inc. and CAS software. The core laboratory also examined protocol compliance in relation to the inclusion criteria. Discrepancies were solved by consensus involving a third investigator.

### Coronary imaging

QCA, OCT, and CAS were performed via 7-Fr guiding catheters. All patients received an intracoronary bolus injection of nitroglycerine (0.2 mg) before each coronary imaging.

For offline analysis, all images were collected using data media, such as DVD. All personal identifying information was removed before downloading to data media.

OCT examination was performed as previously described [11]. Briefly, an optical frequency domain imaging system (FD-OCT), Model C7 Cardiology Imaging System (LightLab Imaging/St. Jude Medical, Westford, Massachusetts, USA) combined with a 0.014-in. conventional angioplasty guidewire was used in this study. After a Z-offset adjustment, the OCT image catheter was advanced to the distal end of the target lesion. Using an injection pump, contrast media at 37 °C was infused through a 6-Fr guiding catheter at 3–4 ml/s for approximately 3 to 4 s. When a blood-free and clear image, which visualized the stent or scaffold cross section, was observed, the OCT imaging core was pulled back at a rate of 20 mm/s using standalone electronic control of the pullback motor.

CAS examination was performed using a balloon occlusion type of angioscopy device (FULLVIEW NEO; iHeart Medical Co.Ltd, Tokyo, Japan) as previously described [12]. Before observation, the white balance was adjusted for color correction. The light power was adjusted to avoid refraction and to determine the color of the plaque. The angioscopic fiber was placed distal to the stent/scaffold and was pulled back manually, from distal to proximal segment of the stent, under careful angioscopic and angiographic guidance. Room-temperature lactated Ringer’s solution was continuously irrigated through the 7Fr delivery catheter at a rate of 0.8–1.0 ml/s by a power injector, and the occlusion balloon was gradually hand-inflated until the image was viewed. When the field of view was flushed clear of blood, inflation of the occlusion balloon was constantly maintained. The guiding catheter pressure, ST-segment changes, cardiac rhythm, and patient comfort were monitored continuously during angioscopy. Each angioscopic image acquisition took about 15–25 s, and all sequences were recorded for subsequent off-line analysis.

### OCT quantitative and qualitative analysis

To assess vascular healing, FD-OCT image analyses were performed with the OCT offline software (LightLab Imaging/St. Jude Medical) at the independent OCT core laboratory (Wakayama Medical University, Wakayama, Japan) by two expert researchers who were blinded to the clinical presentation and lesion characteristics. Through visual screening for all contiguous frames, neointimal thickness, volume and coverage, degree of apposition and all neointimal metrics were evaluated. Degree of stent/scaffold apposition and neointimal coverage on each stent strut cross-section was classified into one of the following categories: (1) well-apposed and covered struts, (2) well-apposed and exposed struts, (3) malapposed and covered struts, (4) malapposed and exposed struts. When the strut/scaffold was covered with neointima, the neointima area was measured as the difference between the intra stent/scaffold area and the lumen area. Neointimal volume was calculated by area per unit × number of slices. Malapposition was defined as present if the measured distance between the center reflection of the strut/scaffold and the vessel wall was greater than the actual stent/scaffolds’ thickness + 20 μm.

For neointimal morphological qualitative analysis, the neointimal tissue was labeled as either homogeneous, heterogenous, or layered, as reported by Gonzalo [13].

The intrastent thrombus and neoatherosclerosis within neointima, together with the presence of lipidic plaque and/or calcification, microvessels, thin-cap fibroatheroma (TCFA), and neointimal rupture were also evaluated. Thrombus was identified as a mass protruding beyond the stent/scaffold strut into the vessel lumen with significant attenuation behind mass.

### CAS qualitative analysis

The angioscopic results were separately reviewed by two experienced angioscopists, who were unaware of either the angiographic or clinical findings at an independent CAS core laboratory (Kurume University, Fukuoka, Japan). There was no interobserver variability, as the angioscopic findings were identical between the two observers.

Angioscopic images were evaluated with a focus on the following: (1) the dominant grade of neointimal coverage (NIC), (2) existence of thrombus, and (3) existence of yellow plaque over and underneath the stent and maximum yellow plaque grade. NIC was classified into four semiquantitative grading categories as: grade 0, stent struts exposed (similar to immediate post implantation); grade 1, struts protruding into the lumen, although covered; grade 2, struts embedded in the neointima, but still translucent; and grade 3, struts fully embedded and invisible. NIC was classified into four grades as previously described [12, 14–16]. If various grades were seen in the stent, the dominant pattern in the entire stent was used as the grade of the stent. Thrombi were defined as masses of red, white or of both colors that adhered to the intima and protruded into the lumen. Yellow plaque grade was determined by visual evaluation as: grade 0, white; grade 1, light yellow; grade 2, medium yellow; and grade 3, intensive yellow. Erosion-like intima (ELI) were determined as a combination of yellow plaque and thrombus with irregular surface.

### Statistical analysis

Continuous variables are presented as the mean ± SD or median. If the data were normally distributed, the Student's paired *t* test was used to compare two groups. Categorical variables were analyzed with Fisher’s exact test for 2 × 2 comparisons. *P* values < 0.05 were considered statistically significant. All analyses were performed using the SAS system version 9.4 (SAS Institute, Cary, NC).

## Results

### Baseline patient and lesion characteristics

A total of 13 eligible patients were enrolled in this ENHANCE study. Twelve patients (BP-EES 13 and BVS 17), since one dropped out due to refusal of informed consent, underwent OCT and CAS evaluation at 4-month angiographic follow-ups. The baseline patient and lesion characteristics are summarized in Tables 1 and 2. All the patients continued dual anti platelet therapy for more than 4 months. The baseline lesion characteristics were similar between the two groups, although bifurcation lesions were more frequently identified in the BP-EES group. All the lesions in these two groups were AHA/ACC type B2/C lesions, and average lesion length was longer than 30 mm. The procedural characteristics were as seen in Table 3, and were compatible in both groups.

**Table 1:** Patient’s baseline characteristics

Variable	Patients (*n* = 12)
Age (years)	64.1 ± 7.1
Male, *n* (%)	7 (58.3)
STEMI, *n* (%)	3 (25)
NSTEMI, UAP, *n* (%)	5 (41.7)
Stable angina, *n* (%)	4 (33.3)
Hypertension, *n* (%)	9 (75.0)
Dyslipidemia, *n* (%)	12 (100)
Diabetes mellitus, *n* (%)	9 (75.0)
Insulin users, *n* (%)	0 (0)
Current smoking, *n* (%)	1 (8.3)
Past smoking, *n* (%)	1 (8.3)
LVEF (%)	64.0 ± 11.4
Serum Cr (μmol/L)	82.2 ± 17.4
Hemodialysis, *n* (%)	0 (0)
Aspirin, *n* (%)	12 (100)
P2Y12 inhibitor, *n* (%)	12 (100)
Clopidogrel	11 (91.7)
Ticagrelor	1 (8.3)
Statin, *n* (%)	12 (100)
RAS inhibitor, *n* (%)	6 (50.0)
β-Blocker, *n* (%)	4 (33.3)

Data are presented as mean ± SD or number (%)

*STEMI* ST-elevation myocardial infarction, *NSTEMI* non-ST-elevation myocardial infarction, *UAP* unstable angina pectoris, *CAD* coronary artery disease, *LVEF* left ventricular ejection fraction, *Cr* creatinine, *RAS* Renin-Angiotensin System

**Table 2:** Baseline lesion characteristics

	BP-EES (*n* = 12)	BVS (*n* = 12)	*P* value
Number of lesions	13	17	
Target vessel, *n* (%)			
LAD	3 (25.0)	5 (41.7)	0.102
LCX	8 (66.7)	3 (25.0)	
RCA	1 (8.3)	4 (33.3)	
Bifurcation, *n* (%)	9 (75.0)	3 (25.0)	0.039
Eccentric, *n* (%)	9 (75.0)	4 (33.3)	0.095
Bend (degree)	23.3 ± 15.7	32.5 ± 20.7	0.236
Type B2/C lesions, *n* (%)	12 (100)	10 (83.3)	0.478
RVD (mm)	2.60 ± 0.57	2.69 ± 0.30	0.639
MLD (mm)	0.95 ± 0.53	1.02 ± 0.35	0.695
DS (%)	65.3 ± 13.0	62.0 ± 13.2	0.541
Lesion length (mm)	33.7 ± 7.3	35.4 ± 15.9	0.737

Data are presented as mean ± SD or number (%)

*BP-EES* bioresorbable polymer-coated everolimus-eluting stents, *BVS* bioresorbable vascular scaffolds, *LAD* left anterior descending artery, *LCX* left circumflex artery, *RCA* right coronary artery, *RVD* reference vessel diameter, *MLD* minimal lumen diameter, *DS* diameter stenosis

**Table 3:** Procedural characteristic and quantitative coronary angiography data

	BP-EES (n = 12)	BVS (n = 12)	*P* value
Index procedural characteristics			
Pre-dilation, *n* (%)	10 (83.3)	12 (100)	0.478
Post-dilation, *n* (%)	11 (91.7)	12 (100)	1.000
Overlapping devices, *n* (%)	1 (8.3)	3 (33.3)	0.317
Post procedure			
Technical success TIMI 3, *n* (%)	12 (100)	12 (100)	N/A
Post balloon diameter (mm)	3.21 ± 0.50	3.21 ± 0.35	0.988
Post balloon pressure (mmHg)	18.2 ± 2.86	19.3 ± 3.26	0.332
Total Stent/Scaffold diameter (mm)	2.87 ± 0.42	3.09 ± 0.32	0.123
Total Stent/Scaffold length (mm)	31.6 ± 10.3	32.2 ± 16.2	0.917
Quantitative coronary angiography data			
Post procedure			
MLD (mm)	2.00 ± 0.44	2.00 ± 0.40	0.989
DS (%)	12.7 ± 9.06	14.67 ± 7.80	0.568
4 M follow-up			
MLD (mm)	2.16 ± 0.39	1.85 ± 0.17	0.144
In-stent LLL (mm)	-0.16 ± 0.33	0.15 ± 0.42	0.056
DS (%)	16.9 ± 2.25	29.1 ± 16.8	0.038
12 M follow-up			
MLD (mm)	1.79 ± 0.56	1.68 ± 0.45	0.614
In-stent LLL (mm)	0.20 ± 0.46	0.40 ± 0.60	0.390
DS (%)	22.5 ± 13.2	37.5 ± 13.1	0.015

Data are presented as mean ± SD or number (%)

*TIMI* thrombolysis in myocardial infarction, *MLD* minimal lumen diameter, *LLL* late lumen loss, *DS* diameter stenosis, *N/A* not available

Eleven patients received a 12 months follow-up, since one patient passed away due to retroperitoneal bleeding at a 4 months follow-up. The clinical results showed that ischemic-driven target lesion revascularization (TLR) was 0 (0%) and 1 (9.1%) in the BP-EES group at 4 and 12 months, respectively, and for BVS group ischemic driven TLR was 2 (16.7%) and 2 (18.2%) at 4 and 12 months, respectively. One of the BP-EES patients developed late stent thrombosis at 12 months and 15 months. This patient presented with STEMI, and on both occasions were found to be non-adherence to DAPT a few weeks before stent thrombosis. One TLR was observed in the BVS group between 12 and 36 months, but no other MACE was observed.

### QCA quantitative analysis

Measurement of baseline, post-procedure, and follow-up QCA data are shown in Table 3. There were no statistical differences between BP-EES and BVS pre-intervention. In the follow-up period, minimum lumen diameter by QCA, after BVS implantation, tended to be smaller than that with BP-EES because percent diameter stenosis with BVS was significantly higher than BP-EES. Late lumen loss at 12 months follow-up showed little difference between BP-EES and BVS.

### OCT analysis

**Fig. 1:**
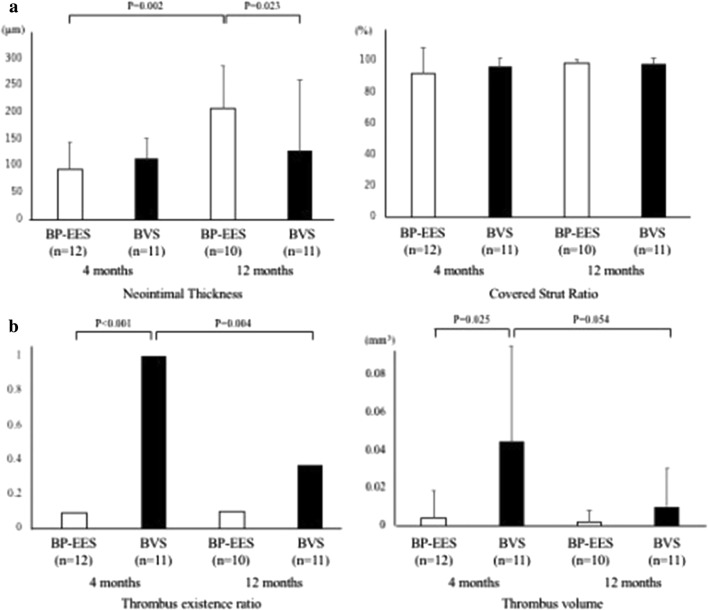
**a** Change from 4 to 12 months in neointimal thickness and covered strut ratio by quantitative OCT analysis. **b** Change from 4 to 12 months in thrombus volume by quantitative OCT analysis. *BP-EES* bioabsorbable polymer-coated everolimus-eluting stents, *BVS* bioresorbable vascular scaffolds

At 4 months, 12 patients underwent OCT and CAS evaluation. In one patient with two BVS, the OCT and CAS catheter could not pass through the target lesion at the 4 months follow-up. 3259 BP-EES struts and 3081 BVS scaffolds were analyzed at 4 months. Planar and volumetric quantitative analysis is presented in Table 4. More than 90% of struts and scaffolds were covered in both groups. Also, there were few malapposed struts to be seen, which also was not statistically different. Besides, there was not any significant difference in neointimal thickness, neointimal volume, and lumen volume between the two groups at 4 months. Intra-stent thrombus was found in only one case in the BP-EES group. However, thrombus was detected in all 12 cases in the BVS group, which showed statistical significance. Although overall thrombus volume was small in both groups, the thrombus volume in the BP-EES group was significantly less than that of the BVS group (Fig. 1).

**Table 4:** Quantitative and qualitative OCT analysis and qualitative CAS analysis

	BP-EES	BVS	*P* value
Quantitative OCT analysis			
4 M follow-up	*n* = 12, 13stents	*n* = 11, 15scaffolds	
Analyzed struts, mean ± SD	271.6 ± 83.8	280.1 ± 132.4	0.858
Minimum lumen area (mm^2^)	3.97 ± 2.01	3.45 ± 1.24	0.456
Minimum stent/BVS area (mm^2^)	4.55 ± 1.83	3.97 ± 1.08	0.366
Maximum neointima area (mm^2^)	1.36 ± 0.51	1.48 ± 0.71	0.648
Thrombus, *n* (%)	1 (8.3)	11 (100)	< 0.001
Thrombus volume (mm^3^)	0.004 ± 0.01	0.045 ± 0.05	0.025
Lumen volume (mm^3^)	158.2 ± 50.7	155.6 ± 90.2	0.936
Stent/BVS volume (mm^3^)	180.0 ± 50.2	176.8 ± 102.2	0.927
Neointimal volume (mm^3^)	22.2 ± 15.2	23.2 ± 15.7	0.875
Mean neointimal thickness (μm)	93.7 ± 49.6	113.5 ± 38.0	0.294
Malapposed struts rate (%)	0.67 ± 1.56	0.09 ± 0.30	0.233
Covered struts rate (%)	91.7 ± 17.1	96.4 ± 5.21	0.379
12 M follow-up	*n* = 10, 10 stents	*n* = 11, 14 scaffolds	
Analyzed struts, mean ± SD	261.3 ± 84.9	280.8 ± 167.8	0.738
Minimum lumen area (mm^2^)	3.27 ± 2.17	3.59 ± 1.08	0.673
Minimum stent/BVS area (mm^2^)	4.74 ± 2.10	4.50 ± 0.94	0.740
Maximum neointima area (mm^2^)	2.74 ± 0.99	1.78 ± 0.85	0.026
Thrombus, *n* (%)	1 (10.0)	4 (36.4)	0.311
Thrombus volume (mm^3^)	0.002 ± 0.01	0.01 ± 0.02	0.221
Lumen volume (mm^3^)	153.1 ± 99.1	140.7 ± 88.1	0.761
Stent/BVS volume (mm^3^)	205.1 ± 100.2	166.4 ± 109.6	0.397
Neointimal volume (mm^3^)	52.1 ± 24.4	28.6 ± 26.0	0.042
Mean neointimal thickness (μm)	206.2 ± 81.2	128.2 ± 61.5	0.023
Malapposed struts rate (%)	0.30 ± 0.67	1.58 ± 3.55	0.244
Covered struts rate (%)	98.8 ± 2.60	97.7 ± 4.17	0.476
Qualitative OCT analysis			
4 M follow-up	*n* = 12, 13 stents	*n* = 11, 15 scaffolds	
Homogeneous, *n* (%)	9 (75.0)	7 (63.6)	0.816
Heterogeneous, *n* (%)	2 (16.7)	3 (27.3)	
Layered, *n* (%)	1 (8.3)	1 (9.1)	
High intensity, *n* (%)	9 (75.0)	8 (72.7)	0.901
Low intensity, *n* (%)	3 (25.0)	3 (27.3)	
12 M follow-up	*n* = 10, 10 stents	*n* = 11, 14 scaffolds	
Homogeneous, *n* (%)	7 (70.0)	8 (72.7)	0.9919
Heterogeneous, *n* (%)	2 (20.0)	2 (18.2)	
Layered, *n* (%)	1 (10.0)	1 (9.1)	
High intensity, *n* (%)	9(90.0)	10(90.9)	0.944
Low intensity, *n* (%)	1(10.0)	1(9.1)	
Qualitative CAS analysis			
4 M follow-up	*n* = 12, 13 stents	*n* = 11, 15 scaffolds	
NIC, grade ± SD	1.76 ± 0.72	N/A	
Thrombus (%)	23.1	93.3	< 0.001
Yellow plaque (%)	84.6	100	0.206
Yellow plaque, grade	0.62	1.333	0.131
Low grade (0–1) (%)	92.3	60.0	
High grade (2–3) (%)	7.7	40.0	
ELI (Erosion-like intima) (%)	15.4	93.3	< 0.001
12 M follow-up	*n* = 10, 9 stents	*n* = 10, 14 scaffolds	
NIC, grade ± SD	2.78 ± 0.44	N/A	
Thrombus (%)	10.0	57.1	0.033
Yellow plaque (%)	40.0	78.6	0.092
Yellow plaque, grade	0.40	1.07	0.110
Low grade (0–1) (%)	90.0	78.6	
High grade (2–3) (%)	10.0	21.4	
ELI (Erosion-like intima) (%)	0.0	57.1	0.006

Data are presented as mean ± SD or number (%)

*NIC* dominant neointimal coverage

Data analyzed 2613 BP-EES struts and 3280 BVS scaffolds at 12 months follow-up revealed that more than 97% of struts were covered by neointima in both groups (Table 4). The neointimal thickness and volume were significantly higher in the BP-EES group than the BVS group, although lumen volume was equivalent in the two groups.

At 4–12 months follow-up, changes in neointimal thickness in the BP-EES group increased significantly, but not in the BVS group. During this observation period, thrombus decreased significantly in the BVS group, and there was no difference in the observation frequency of thrombi at 12 months, although a significant difference was observed at 4 months. The qualitative OCT analysis in both groups showed no significant differences in neointimal morphological characteristics, and no neoatherosclerosis findings.

### CAS qualitative analysis

Two CAS images in one patient were not evaluated due to incomplete blood clearance at 12 months follow up. The CAS findings at 4 months and 12 months follow-up for both groups are showed in Table 4. Average dominant neointimal coverage grade in BP-EES was estimated semi-quantitatively at both follow-ups. However, it was impossible to estimate the grade of neointimal coverage of BVS because of the lack of definition with BVS-evaluation-criteria by angioscopy. In addition, the polymer-based-scaffold was completely different from metallic struts in angioscopic evaluation and translucent scaffolds are inappropriate for visual estimation.

At 4 months follow-up, thrombi were more frequently observed in BVS than BP-EES. The frequency and grade of yellow plaque was numerically higher in BVS than in BP-EES, but was not significant in either follow-up. The ELI, which, under CAS observation, was determined as a combination of yellow plaque and thrombus with an irregular surface, was significantly higher in BVS at both follow-ups.

**Fig. 2:**
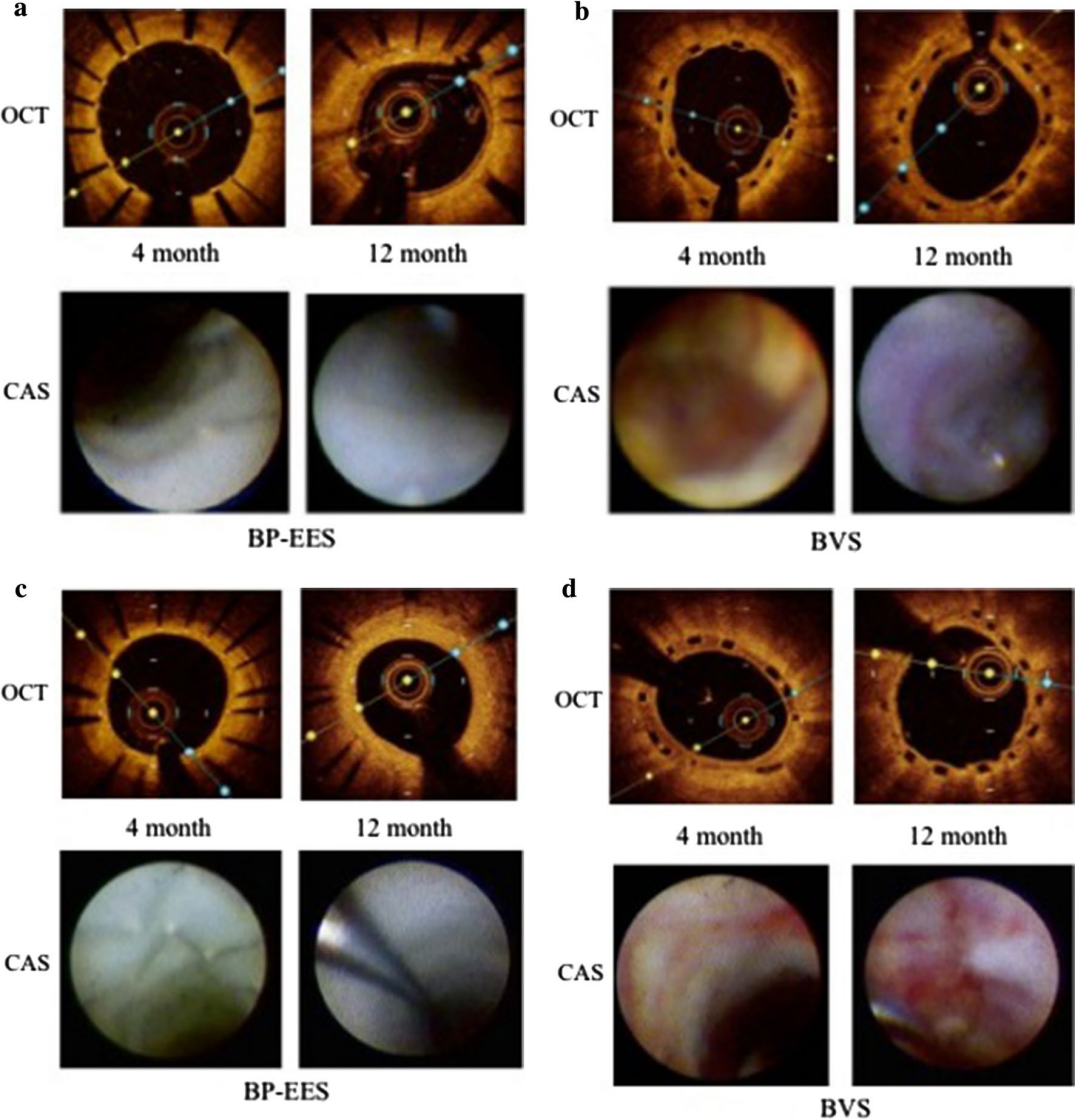
Representative OCT and CAS image at 4 and 12 months. The OCT images in upper panel, and CAS images in lower panel. **a**, **c** Follow-up images at 4 and 12 months after BP-EES implantation. OCT images showed almost all struts of BP-EES were covered at 4 months although the thickness of neointima was thin. CAS images of BP-EES demonstrate white homogenous neointimal coverage (NIC) grade 1–2 at 4 months. A small red intra-mural thrombi (arrow) at proximal edge in **a**. At 12 months, NIC grade was 3, without thrombus in both cases. **b**, **d** follow-up images at 4 and 12 months after BVS implantation. OCT images showed most scaffolds were well covered even at 4 months. At 12 months, the neointimal thickness did not increased. CAS images of BVS revealed severe ELI (erosion-like intima) at 4 months in both cases. At 12 months, ELI was attenuated but still observed. *OCT* optical coherence tomography, *CAS* coronary angioscopy

Representative cases are shown in Fig. 2.

## Discussion

In the ENHANCE study, BVS and BP-EES were implanted at the same time in different coronary arteries of the same patients. The condition in neointimal coverage and healing was observed and compared by OCT and coronary angioscopy at 4 months and 12 months after deployment. As assessed by OCT at 4 months, both BVS and BP-EES struts were well covered (over 97%), and neointimal thickness was not different between BP-EES and BVS. Thrombus formation at the stent and scaffold placement site was overwhelmingly more frequent with BVS. At the 12 months OCT evaluation, the neointimal wall thickness of BP-EES significantly increased compared to 4 months, but no change was observed in the BVS group. Thrombus was detected in only one case in the BP-EES group at 4 months and 12 months. Thrombus volumes were significantly reduced in the BVS group. The frequency in thrombus detection tended to decrease numerically, thus there was no significant difference.

In the qualitative examination of stent placement by angioscopy, the progress of these two stents was completely different. In the thin metal strut BP-EES, the stent struts were observed through the neointima at 4 months, but the translucent white neointima was clearly coated at a high rate. At 12 months after placement, the stent struts were completely buried and were not observed at all. On the other hand, for polymer scaffolds in BVS, a visual evaluation method for the material to be absorbed, such as polymer scaffold, has not been established by angioscope, and conventional methods for grading neointimal coverage could not be adopted. Surface character observation through angioscope was, however, possible, and red thrombi and yellow plaque were observed in multiple locations in almost all the cases at 4 months after BVS implantation. In this context we have created a new term, ELI (erosion-like intima), to describe this condition. At 12 months, the frequency in observation of yellow plaque tended to decrease. Although this phenomenon is considered progress of the healing process in BVS, the fact that ELI was frequently observed in BVS may imply the prolongation of the healing process as compared to BP-EES.

In the endovascular treatment devices with polymer used in this study, the process of polymer absorption up to 4 months is theoretically similar for both, but one is metal-based DES, and the polymer coating on the abluminal surface disappears after 3–4 months. The other is a conglomeration of polymer, where the polymer elution would last for several years. For this reason, a marked difference in the healing process was observed at 4 months, and stable healing was observed in BP-EES, whereas instability in the vascular wall was noticeable in BVS. At 12 months re-evaluation, BP-EES showed a similar increase in neointimal coverage of bare metal stent, and a stable vessel-wall healing process without red thrombi and yellow plaque was observed. On the other hand, BVS was considered to be progressing the healing process of the scaffold placement compared with the healing status observed at the 4th month. However, the intravascular condition was still considered to be unstable.

### Yellow plaque, thrombus and erosion-like intima (ELI)

The characteristics of angioscope are qualitative detection of yellow plaque and thrombi that can be distinguished by color tone, although quantitative evaluation cannot be performed. Many reports showed angioscopically detected yellow plaque has been commonly found at the culprit lesions of the spontaneous acute coronary syndrome; and yellow plaque, especially high intensity yellow plaque, is considered fragile and a high risk for coronary arteries [17, 18].

We previously reported that poor neointimal coverage at 8 months after DES placement was associated with endothelial dysfunction and also with the presence of yellow plaque and thrombi [19]. It has also been reported that endothelial dysfunction after DES implantation lasts more than 2 years [20].

Yellow plaque, thrombosis and endothelial dysfunction are known to be particularly prevalent in the first generation DES, and such delayed arterial healing is considered to be associated with inflammation [21]. Furthermore, it has been reported that yellow plaque observed after DES placement is associated with subsequent cardiac events, and the underlying mechanism is presumed to be the development of arteriosclerosis [22].

The purpose of the current ENHANCE Study is to assess the healing process of two completely different endovascular treatment devices with two imaging modalities, rather than post-implantation events. In OCT, a relatively stable healing process was observed with BP-EES, as in the previous evaluations. In CAS (an angioscope in which the inside of a blood vessel was directly observed like a video camera), it was observed that erosion-like intima (ELI) was significantly higher in BVS and this was considered to be indicative of intravascular instability after placement. We believe that the present results lead to a mechanism for the large-scale clinical trials of BVS to be unsuccessful.

### Study limitations

This OCT and CAS section of the ENHANCE study has the following limitations. First, this was a relatively small sample-size, non-randomized single center study. Second, the pre-PCI OCT and CAS findings were not evaluated. Third, the current coronary angioscope technology provides a forward-viewing system only and the viewing range is limited. In contrast, OCT provides high resolution cross-sectional imaging of the entire vessel, which may offer superior quantitative evaluation. However, at the present time, it is not possible, even with OCT, to discriminate thin neointimal tissue from fibrin and thrombus. Finally, the later stages of vessel healing remain unknown. Further studies with serial long-term follow-ups are required to evaluate the relationships between early vascular healing, future major adverse events and clinical performance.

## Conclusion

Between 4 and 12 months follow-up observation via OCT and angioscopy, BP-EES showed early healing and stable intra-stent conditions. However, the healing process of BVS was not completed untill the 12th month.
